# Injectable Hydrogels for Improving Cardiac Cell Therapy—In Vivo Evidence and Translational Challenges

**DOI:** 10.3390/gels7010007

**Published:** 2021-01-22

**Authors:** Cecilie Hoeeg, Alireza Dolatshahi-Pirouz, Bjarke Follin

**Affiliations:** 1 Cardiology Stem Cell Centre, Rigshospitalet, Henrik Harpestrengs Vej 4C, 2100 Copenhagen, Denmark; cecilie.hoeeg.pedersen@regionh.dk; 2 Department of Health Technology, Center for Intestinal Absorption and Transport of Biopharmaceuticals, Technical University of Denmark, 2800 Kongens Lyngby, Denmark; aldo@dtu.dk; 3 Radboud University Medical Center, Radboud Institute for Molecular Life Sciences, Department of Dentistry—Regenerative Biomaterials, Philips van Leydenlaan 25, 6525EX Nijmegen, The Netherlands; 4 Department of Immunology and Microbiology, University of Copenhagen, Blegdamsvej 3, 2200 Copenhagen, Denmark

**Keywords:** hydrogel, cell therapy, regenerative therapy, cardiac disease, heart failure, mesenchymal stem cell, delivery

## Abstract

Cell therapy has the potential to regenerate cardiac tissue and treat a variety of cardiac diseases which are currently without effective treatment. This novel approach to treatment has demonstrated clinical efficiency, despite low retention of the cell products in the heart. It has been shown that improving retention often leads to improved functional outcome. A feasible method of improving cell graft retention is administration of injectable hydrogels. Over the last decade, a variety of injectable hydrogels have been investigated preclinically for their potential to improve the effects of cardiac cell therapy. These hydrogels are created with different polymers, properties, and additional functional motifs and differ in their approaches for encapsulating different cell types. Only one combinational therapy has been tested in a clinical randomized controlled trial. In this review, the latest research on the potential of injectable hydrogels for delivery of cell therapy is discussed, together with potential roadblocks for clinical translation and recommendations for future explorations to facilitate future translation.

## 1. Introduction

Cardiovascular diseases remain the leading cause of death worldwide and include pathological disorders such as acute myocardial infarction (AMI) and heart failure [1]. Current treatment options focus primarily on slowing disease progression and ameliorating symptoms, but therapies that regenerate or reverse myocardial remodeling have yet to be developed and/or implemented in clinical practice [2]. Therefore, several patients with end-stage heart failure will develop a need for heart transplantation, a treatment highly restricted by donor availability.

For almost two decades, cell therapy has been investigated for treatment of several cardiovascular diseases. Various cell types have been tested in clinical trials, and many types have been investigated in preclinical studies. Several cell types have been demonstrated to have a good clinical safety profile and to be capable of ameliorating or reversing the myocardial remodeling [2]. Cell therapy may very well be groundbreaking in treatment of otherwise non-treatable conditions, including several heart diseases.

Though the new cell therapy has promising perspectives, it has limits. Already in 2011, Malliaras and Marban acknowledged that the low retention in the heart was a hindrance for the effectiveness of cardiac cell therapy [3]. In the same paper published a decade ago, the authors mention that studies using different regenerative cell types found a good correlation between cardiac retention rates and efficiency of the treatment and concluded that improving physical retention may hold the key to unlocking the potential of cell therapy for cardiological diseases. The following year, Mathieu et al. found an improvement of cardiac cell therapy by administering the cells in an injectable hydrogel [4].

During the last decade, several approaches for improving the retention of cell therapy in the heart have been tested. Some of the cell loss is explained by migration via the lymphatic system [5]. This could be improved by tuning the cell products by overexpressing adhesion and integration of surface proteins. However, even if the cell product is more prone to adhere and integrate in the environment, the physical pressure from the contracting heart, as well as the hostile inflammatory environment, will still lead to expulsion and immune system activation [6,7]. This calls for a technology providing better retention in the tissue as well as shielding of the cell product. Since cell therapy products need to interact with the endogenous cell populations, a certain degree of porosity is essential in addition to the retention and shielding. All of this is a perfect fit for hydrogels. Since the publication by Malliaras and Marban, the many attempts to enhance cardiac cell therapy using biomaterials, including hydrogels, have resulted in hundreds of preclinical studies and the first clinical randomized controlled trial being published in 2020 [6,8].

This review describes the potential functional benefit of using injectable hydrogels for cell therapy administration. Furthermore, the different cell and hydrogel types as well as roadblocks for clinical translation are elucidated. Due to our focus on large-scale clinical application, non-injectable scaffolds or cardiac patches are not within the scope of this review.

## 2. Injectable Hydrogels

In simple terms, hydrogels are highly hydrated biomaterials that enable 3D encapsulation of living cells. The high water content enables an efficient exchange of nutrients and metabolic waste products with the surrounding environment, while providing the encapsulated cells with a hydrated 3D and immune protective environment. Hydrogels can also be fine-tuned to yield mechanical properties similar to the extracellular matrix (ECM) of native tissues and provide cells with native-like biochemical stimuli that can direct them towards desired fates. Importantly, the soft nature of hydrogels makes them ideal candidates for injectable biomaterials capable of carrying cells into pre-destined target sites inside the human body [9]. The fact that these injectable cell carriers can offer protection against the potential compromising mechanical shear forces during the injection phase while providing the injected cells with a well-controlled and retained 3D microenvironment makes them feasible for cardiac cell delivery as well.

### 2.1. Natural Hydrogels

Hydrogels are often divided into natural or synthetic hydrogels. Each group has their own advantages and challenges. Several natural biopolymers have already been approved for clinical application, making the use of these gels a quick choice for translatable cell carriers. Natural hydrogels include collagen, silk fibroin, chitosan, cellulose, alginate, gelatin, and hyaluronate hydrogels [10,11,12,13,14,15,16,17]. The natural polymers are known to be very biocompatible. Most natural hydrogels, including those created from hyaluronate, fibrin, and collagen, are degraded by cellular enzymes, while alginate hydrogels degrade by ion exchange with the surrounding environment. For these reasons, the cross-linking and degradation of unmodified natural hydrogels is considered uncontrollable in vivo but very safe unless the gels are modified [18]. Since some of the natural polymers either are biologically inert or incapable of encompassing the electrical and mechanical properties of the target tissue, ECM engineers have, over the years, developed several methodologies to remedy such shortcomings. A widely used strategy for enhancing the bioactivity of biologically inert hydrogels is through growth factor or arginine–glycine–aspartic acid peptide (RGD) conjugation on the hydrogel backbone [19].

### 2.2. Synthetic Hydrogels

In contrast to natural hydrogels, synthetic hydrogels are highly controllable and customizable. The use of synthetic hydrogels for cell delivery has increased during recent years. The number of synthetic hydrogel pre-polymers used in tissue engineering is quite impressive and rapidly growing. They include various polypeptides and biopolymers expressed by genetically altered cells and bacteria or others such as polyvinyl alcohol (PVA), polyacrylamide, poly(N-isopropylacrylamide) (PNIPAm), polyethylene glycol (PEG), and poly(3,4-ethylenedioxythiophen) polystyrene sulfonate (PEDOT:PSS) made by chemists [20]. Synthetic hydrogels are not considered to be as biocompatible as natural polymers, either due to their origin of synthesis or their cross-linking [9,18]. Many of the synthetic hydrogels are not degraded in vivo unless modified by, i.e., incorporation of natural biodegradable polymers. To circumvent this issue, self-assembling peptide hydrogels such as the RADA hydrogel have been shown to degrade without toxicity of degradation products [21]. From a clinical perspective, only PEG and PVA have, so far, managed to maintain a good reputation among physicians and federal agencies [22].

The choice of hydrogel type for cell delivery should be made carefully, as hydrogel properties affect the incorporated cells. Consequently, the choice should not be guided by preference but rather by impact on the cells used for the treatment and the tissue environment and administration options in the clinical indication.

## 3. Hydrogel Properties and Delivery Depends on Clinical Indication

### 3.1. Acute Myocardial Infarction

So far, cell therapy has been shown to have little effect in patients with acute myocardial infarction (AMI), despite a body of preclinical evidence showing the opposite [23,24]. This has most recently been shown in the Phase III autologous bone marrow cell therapy in acute myocardial infarction trial (BAMI) [25]. The investigators concluded that the improvement in the standard of care for AMI patients is simply so good that it requires too many patients, close to 10,000, to demonstrate a potential effect of cell therapy on hard end-points such as death. As an important note, this trial did not meet the number of recruited patients and ended up including 375 out of the planned 3000 [25]. Since this is caused by the control group, the results will most likely be the same across all cell therapies for AMI. As such, if cell therapy is to be effective in AMI, the current clinical approach needs to be significantly improved. A feasible method of doing this while retaining the workflow optimized over the last 10 years of cell therapy would be administration of the cells in injectable hydrogels.

If researchers plan to apply hydrogels for cell delivery in AMI, it is important to be aware of the myocardial environment following AMI. The extensive inflammation and apoptosis after AMI result in the peri-infarct myocardium being relatively soft [26]. This makes it difficult to inject cells by transendocardial stem cell injection (TESI), though this administration has been shown to be superior in terms of cell retention and clinical efficacy [6,27,28,29]. TESI is performed using a catheter to enter the left ventricle through the venous system. The conductivity of the ventricle can be mapped using the NOGA mapping system, creating a map of the non-conductive infarct area. Clinicians look at the X-ray for TESI injection and are able to map the injection site in the NOGA program. As such, it is possible to deliver cells to the peri-infarct area through minimally invasive methods. However, due to the soft myocardium and ease of access, most cell therapies in AMI have been delivered by intracoronary injection [23]. If this approach is to be continued, the chosen hydrogel for delivery must be able to cross the endothelial barrier and crosslink at the site of injury. Such hydrogels have been developed and applied, taking advantage of AMI-specific tissue attributes such as increased calcium levels [30].

### 3.2. Chronic Ischemic Heart Failure and Non-Ischemic Dilated Cardiomyopathy

In contrast to AMI, cell therapy has been shown to be clinically effective in treatment of chronic ischemic heart failure (CIHF) [23,31]. The myocardium of heart failure patients is not as soft as in AMI patients, making it more feasible to use TESI delivery. Though there is persistent chronic inflammation in CIHF, it is not to the same scale and with as dynamic changes as in AMI [32]. This requires less shielding against the hostile environment caused by the disease, though there will still be an acute immune reaction to the intramuscular injection in itself.

In addition to CIHF, cell therapy shows promising tendencies in non-ischemic dilated cardiomyopathy (NIDCM), with even larger effect size and solid preclinical evidence [33,34]. NIDCM is a disease with varying etiology, including genetic mutations, exposure to certain drugs and toxins, and autoimmune diseases. Despite this variation, common tissue denominators are present in the myocardium, in which the myocardial microenvironment is characterized by fibrosis, chronic inflammation, oxidative stress, and dysfunctional vessels [28]. Consequently, survival and engraftment of transplanted cells might be optimized by shielding the cells from the harsh microenvironment, thus improving the treatment effect. However, there are currently no studies on hydrogel-delivered cell therapy in NIDCM [34].

As with CIHF, the cardiac tissue in NIDCM is less soft and porous, making TESI injections feasible. As TESI is currently applied in several clinical trials with cell therapy, designing a hydrogel for this purpose may ease the translation into the clinic [28,35,36]. Though TESI has proven to be the best current option for cell delivery, it poses a challenge for cell delivery in hydrogels. A common use of hydrogel for cell delivery is to apply it as an injection medium for the cells—this will be referred to as bulk delivery. However, during TESI, the catheter is inside the patient for up to an hour, and the hydrogel–cell mixture is exposed to body temperature through this catheter in the minutes leading to injection. This makes the use of hydrogels with temperature-dependent cross-linking difficult. In addition, TESI currently occurs through a single barrel, which is a challenge for quickly cross-linking hydrogels [37].

Besides delivery methods, optimal hydrogel design may differ between diseases. As mentioned above, hydrogels have been developed for in-situ cross-linking due to higher calcium levels in AMI. However, for both CIHF and NIDCM, the amount of myocardial apoptosis and, therefore, release of calcium is lower compared to AMI. This means that hydrogels designed for calcium-dependent in-situ cross-linking may be limited to application in AMI [38].

### 3.3. Coronary Artery By-Pass Graft

In addition to the hypoxic and inflammatory environment in the failing heart, coronary artery bypass grafting (CABG) also introduces oxidative stress when blood supply to the ischemic myocardium is re-established [39]. This calls for additional shielding of the applied cell therapy products, regardless of cell type. Cell-containing injectable hydrogels have been applied clinically during CABG, where they can be injected with great precision during open chest surgery [8]. This makes the issues of TESI delivery obsolete.

Open-chest operations are open for other biomaterial applications such as scaffolds or cell sheets. As mentioned earlier, non-injectable scaffolds or cell sheets are not included in this review as these biomaterials utilize a different technology and could be discussed in length in a separate review. Most of the patients treated with cell therapy for the above-mentioned indications will not receive CABG. For large-scale treatment of such patients, it is unlikely that an open-chest procedure will be the standard of care in the long term.

## 4. Cell Therapy Candidates and Hydrogel Requirements

The approaches to cell therapy for cardiac regeneration can generally be divided into two categories. First, to boost endogenous repair mechanisms. Most adult stem cell therapies including mesenchymal stromal cells (MSCs) and derived paracrine mediators fall into this category. This may also simply be called cell therapy and assisted cell therapy if hydrogels or other carriers are used. Second, to provide new contracting cells. Pluripotent stem cell-derived cardiomyocytes, myoblasts, and cardiopoietic cells can be included into this category. The application of such an ex vivo generated construct is also called tissue engineering. Cardiac tissue engineering approaches include ex vivo generated scaffolds or cell sheets combined with contracting cell types or cells differentiating into contracting cells. The term also includes the application of injectable hydrogels with these cells. Some cell types fall into both categories, and therefore, the treatment is both cell therapy and tissue engineering.

### 4.1. Mesenchymal Stromal Cells and Endothelial Progenitor Cells

For cardiac regeneration, mesenchymal stromal cells (MSCs) are the furthest in clinical development [2]. These cells are relatively easily harvested and cultured for clinical use [5]. The technology is more straightforward compared to more potent cell types, such as induced pluripotent stem cells (iPSCs) or embryonic stem cells (ESCs), as well as differentiated cell types from pluripotent cells. Due to the limited proliferation and differentiation potential of MSCs, there are less safety issues. This has facilitated their quick translation into clinical trials, providing a bulk of evidence for the safety of these cells in cardiac indications [23,28].

Initially, MSCs were thought to transdifferentiate into cardiomyocytes, directly aiding the pump function of the damaged heart [2]. However, overwhelming evidence has proven that MSCs mainly exert their regenerative functions through paracrine interaction with the surrounding cells. This can lead to immunomodulation, anti-apoptosis, and anti-fibrosis [32,40]. As such, MSC-derived secretions have been tested as cell-free alternatives to cell therapy. Results comparing the effect of MSCs and MSC-derived secretions on cardiac function are, however, inconsistent, though pointing towards cell therapy being superior [41]. Common for these approaches is that they initiate an endogenous wound healing response, including angiogenesis, activation of macrophage subtypes, and homing of circulating stromal cells [2].

Endothelial progenitor cells (EPCs) are derived from bone marrow or blood and are thought to function much like MSCs. Like MSCs, they can be harvested and cultured with relative ease, and they are deemed safe due to function as progenitors [2].

Since MSC and EPC therapy is thought to be mostly paracrine, the main goal of combining this therapy with hydrogels is to provide a sustained release of the paracrine mediators. As such, complete functional integration is not required, but porosity for paracrine interaction is. A great strength of MSCs is their ability to adapt to the environment and respond accordingly. This can be illustrated by their ability to be “licensed” by exposing them to inflammatory mediators such as interferon-γ, which in turn boosts their immunomodulatory abilities [42]. Therefore, for optimal utilization of MSC therapy, the cells need to have environmental cues to respond to, and the hydrogels will have to be permeable for these environmental cues.

New evidence suggests that the cardiac regenerative effect of MSC therapy occurs through an induction of sterile inflammation [7]. The downstream effect of this sterile inflammation could also be caused by engulfment or induced apoptosis of the MSCs by the endogenous immune system [43]. This could explain why the therapy has a functional effect despite low engraftment. If this physical interaction is indeed needed for optimal therapy, the hydrogel must eventually release the encapsulated cells. Due to the high water content and biodegradability of most applied hydrogels, the cells usually sediment from the gel or are released as the gel slowly dissolves, allowing the physical interaction to occur as the cells are slowly released [10,44].

### 4.2. Cardiopoietic Cells and Cardiomyocytes

An alternative to MSCs are cardiac progenitor cells (CPCs) and pluripotent stem cell-derived cardiomyocytes [45]. In contrast to MSCs, CPCs and differentiated cardiomyocytes may contribute by myocardial engraftment and subsequent contracting power. At present, three ways of sorting CPCs have been successfully applied for patients with cardiovascular disease: c-kit+ sorted cells, spheroid selection of cardiosphere-derived cells, and Sca1+ sorted cells. Despite different sorting criteria, they possess almost identical transcription profiles, suggesting that they have similar regenerative properties [46]. Recent evidence suggests that CPCs can replenish adult injured cardiomyocytes and vascular cells through differentiation. As is the case for MSCs, paracrine secretion is, likewise, a pivotal regenerative mechanism exerted by CPCs. Proteomic analysis of the CPC secretome revealed a complex profile of both proteins, humoral factors, and molecules, which has the potential to stimulate differentiation of endogenous stem cells and modulate angiogenic, fibrotic, apoptotic, and immunological processes [46,47,48].

Differentiated cardiomyocytes can be generated from ESCs or iPSCs and constitute another alternative for cell transplantations. The potential of differentiated cardiomyocytes primarily relies on their ability to electromechanically couple with the viable host myocardium by forming gap junctions with neighboring cells. Furthermore, they maintain a sarcomeric phenotype in vivo and possess contractile properties, thus improving systolic function. Despite the potential, delivery of differentiated cardiomyocytes has been shown to halt the deterioration of systolic function but has failed to improve the already diminished systolic function. Furthermore, application of this cell type entails the same obstacles including poor retention and engraftment. Therefore, novel cell vehicles for localized cell delivery should be explored.

## 5. Current In Vivo Evidence

### 5.1. Clinical Studies

To date, three clinical studies have combined cell therapy and hydrogels (Table 1). All treatments were performed during CABG. Though open-chest operations will not be applicable for all patients as mentioned earlier, CABG is a good indication to test these combination applications due to the low pump function of the heart and visibility of the application area. Chachques et al. injected autologous bone marrow mononuclear cells into the infarct of 15 patients during CABG [49]. The injection sites were sealed with two layers of collagen I scaffolds, with the scaffold closest to the injection being embedded with mononuclear cells. The combination treatment was deemed safe. Menasché et al. administered human ESC-derived cardiovascular progenitor cells in a fibrin patch in six patients during CABG [50]. The method proved safe in terms of off-target tumor formation and arrhythmias, while three patients developed alloimmunization. Finally, He et al. recently performed the first randomized controlled trial using cell therapy and hydrogels on patients receiving CABG [8]. Eighteen patients were injected with allogeneic umbilical cord-derived MSCs (UC-MSCs) embedded in a collagen hydrogel during CABG, 17 patients were treated with UC-MSCs only during CABG, and 15 patients only received CABG. The treatment was safe and the combination of UC-MSCs in the collagen hydrogel resulted in significantly lower scar size percentage 12 months after the treatment compared to the other groups. When focusing on the patients with a baseline left ventricular ejection fraction (LVEF) below 40.0%, mean LVEF changes after 3, 6, and 12 months were 9.14%, 9.84%, and 9.35% in the UC-MSCs in collagen hydrogel, 3.38%, 3.39%, and 6.59% in the UC-MSC group, and 4.17%, 4.40%, and 3.62% in the control group, respectively. The LVEF was significantly increased at all follow-up time points for the UC-MSCs in collagen hydrogel, while it was only significant at the 12 month follow-up for the UC-MSC group. He et al. were the only study group to include a cell-only group, making this the first clinical evidence of potential benefit of administering cell therapy in a hydrogel [8].

### 5.2. Preclinical Studies

Most studies comparing the in vivo effect of cells administered in saline with cells administered in hydrogels report an improved beneficial effect in the hydrogel group (Table 2). Furthermore, the majority of recent studies report cell therapy to have a positive effect on LVEF and a larger effect size when administering cells in hydrogel compared to the non-treated group [10,12,15,17,51,52,53,54,55,56,57]. Other studies found no benefit of cell therapy alone, but only a functional effect of cells in hydrogels [10,13,14,15,16,48,58,59,60,61,62]. A direct comparison between the cell-only group and the cell in hydrogel found significant functional benefits of administration in the hydrogel [10,12,13,14,15,16,17,48,51,52,53,54,56,57,61,62,63]. A few of the recent studies included both a cell-only control and a hydrogel-only control. For most studies comparing the functional effect of cells-only and hydrogel-only groups with that of the combination treatment, a synergistic effect was observed [10,16,17,52,54,56,57,63]. Few studies reported no improvement with the combination treatment compared to cells only [11,45,64]. Findings include improved pump function measured as LVEF, fractional shortening (FS), or perfusion. This is generally reported together with increased scar thickness, increased vessel density, lower degree of fibrosis, and smaller infarct size. These are the same parameters affected by cell therapy itself and seem to be an enhancement of their known cardiac regenerative properties. For most studies, the enhanced benefit of hydrogel delivery is observed together with enhanced retention of the cells, regardless of cell type. Most studies reporting both retention and function find that these are directly correlated. However, this does not always apply, and there is evidence that the properties of the hydrogels are crucial for achieving enhanced efficacy.

Only few studies have made head-to-head comparisons between different types of hydrogels regarding functional in vivo efficacy. Tan et al. compared the efficacy of hESC-derived cardiomyocytes administered in either Matrigel, alginate, or hyaluronate hydrogels in a rat model of subacute myocardial infarction (MI) [65]. They found that all hydrogel deliveries improved LVEF, but hyaluronate increased LVEF more than Matrigel and alginate. This suggests that some basic hydrogels are better suited for delivery of certain cell types than others. However, hydrogel cell delivery can be applied in different ways and through various routes, and the hydrogels can be tweaked with both peptide integration and in terms of stiffness through the degree of cross-linking, which also affects the outcome.

## 6. Important Hydrogel Properties

### 6.1. Stiffness and Porosity

For cell therapy relying on the differentiation of the applied cells, the mechanical properties of the gels are very important. Since cell fate is hugely affected by substrate stiffness, a stiffness resembling that of cardiac tissue is preferred for differentiation. However, hydrogels commonly applied in this field of research are softer than cardiac tissue [48]. This is most likely to accommodate diffusion of nutrients, cell product secretions, and viability of applied cells [48]. Kanda et al. found that coating explant-derived cardiac stem cells (EDCs) with agarose and polydimethylsiloxane (PDMS) containing fibronectin and fibrinogen only improved the efficacy of EDC therapy in the stiffer version of the hydrogel [61]. Differentiation was not investigated, but the improvements were ascribed to differences in secretory activity. This goes to show that even if the mechanisms of action of the cell product are presumed to be differentiation and integration, it is important that secretion of trophic factors is maintained. As such, enhanced improvement of cardiac function has been obtained with increased differentiation of CPCs when the secretion was maintained, but not when secretion was inhibited [48].

The impact of stiffness on differentiation also applies to the commonly used MSCs [66]. A general observation is that when MSCs start to commit to a certain lineage, their secretion of trophic factors decreases [48]. Keeping the stiffness of the hydrogel from influencing MSC differentiation is therefore key. In addition, it is known that the secretion from MSCs depends on the environment. Therefore, investigating whether MSCs in the hydrogel are capable of responding to environmental cues such as hypoxia, inflammatory stimuli, or even co-cultured cells is important [44]. If a proper hydrogel is chosen, the mere fact that the cells are elevated to 3D culture could increase their paracrine potential [44]. Thus, a first step in designing hydrogels for delivery of secretory cell types should be to investigate in vitro if the applied hydrogels permit proper secretion, allow for cross-talk with the environment, and do not induce unwanted differentiation.

For delivery of extracellular vesicles or secretomes, smaller pore size is required if sustained delivery is to be achieved [56].

### 6.2. Functionalization

Hydrogels have been modified to both improve the activity of the delivered cells as well as to induce myocardial regeneration themselves (Table 2). These properties of hydrogels are considered complementary effects to the ones exerted by the delivered cell product.

#### 6.2.1. Adhesion Motifs

Bhutani et al. found that the same hydrogel encapsulation led to different outcomes [48]. Using VPM cross-linker (GCRDVPMSMRGGDRCG) (New England Peptide) and Maleimide-cross-linked poly(ethylene glycol) solution, they encapsulated human CPCs and either adhesive motifs RGD or the collagen derived GFOGER peptide, or non-adhesive RDG. While the non-adhesive hydrogel significantly enhanced the functional benefit of CPC treatment, this was not the case for the adhesive hydrogels. The functional benefit was correlated with the retention of the cells. Even if the GFOGER adhesive hydrogel had similar mechanical properties as the non-adhesive hydrogel, the outcomes were still different. The GFOGER peptide favored cardiomyocyte lineage commitment, while it abolished the secretion of VEGF, hepatocyte growth factor, and, partially, fibroblast growth factor. Hence, for this setup, the improved attachment and lineage commitment did not result in a functional effect. The functional effect could not be explained purely by paracrine secretion either, since the secretions by the cells were similar in vitro in the RGD and RDG peptide gels.

This emphasizes that there is a complex interaction between the properties of the hydrogels, the incorporated motifs, and the cell product. These interactions need to be understood in order to tailor the hydrogels to the needs of the specific cells used. This may be particularly important with cells such as the C-kit+ CPCs, which fall within the cell therapy categories of both physical cardiomyocyte support and paracrine support.

#### 6.2.2. Angiogenesis

Pro-angiogenic modifications of hydrogels have been achieved through addition of peptides such as SDKP or SVVYGLR. The use of hydrogels with these modifications for MSC delivery improved cardiac function to a greater extent than cells delivered alone and increased myocardial angiogenesis in a rat model of AMI [53,54]. The SDKP modified gel itself even improved cardiac function comparable to the combined treatment of MSCs in SDKP gel [53]. Others have achieved an enhanced angiogenic effect through incorporation of heparin or VEGF into the hydrogel [13,59]. The MSC delivery in these hydrogels led to improved cardiac vessel density and LVEF compared to their respective MSC control groups, suggesting that the angiogenic effects of cell therapy can be enhanced using modified hydrogels.

#### 6.2.3. Graft Survival and Activity

Besides boosting angiogenesis, which has been the primary focus of hydrogel functionalization, studies have tried to enhance cell survival, engraftment, and proliferation [16,48,56]. Gerbin et al. tested a hydrogel with immobilized Notch ligand Delta-1 and hESC cardiomyocytes in an athymic rat model of AMI [16]. The combination of the Notch 1 functionalized hydrogel and hESC cardiomyocytes increased graft size and hESC cardiomyocyte proliferation in vivo as well as improving cardiac function compared to controls. Han et al. evaluated a peptide amphiphile -GHRPS NapFF hydrogel containing human umbilical cordMSC-derived exosomes [56]. The hydrogel/exosome mixture was injected into the MI border zone of rats, which subsequently improved LVEF compared to controls with exosomes or hydrogel alone. Furthermore, the hydrogel improved exosome retention and reduced cardiac inflammation and fibrosis. Choe et al. focused on protecting human UC-MSCs from oxidative stress by microencapsulating them in graphene oxide alginate. Further protection was generated by reducing the hydrogel. While the authors found no significant advantage of hydrogel delivery in terms of LVEF changes compared to cells only, the reduced graphene alginate significantly decreased the fibrosis area and increased the thickness of the left ventricle compared to the other groups [64].

### 6.3. Retention is Not Everything

Most studies reporting both retention and efficacy find the retention of the administered cells to be directly correlated with treatment effect (Table 2). Opposing this, Kanda et al. found a less-stiff single EDC cell hydrogel encapsulation to result in increased retention, but not increased functional effect, when compared to non-encapsulated EDCs. Another formulation of the hydrogel leading to greater stiffness resulted in similarly improved retention but also improved treatment effect. A comparison between the paracrine activity of EDCs in the different gels showed that the stiffer gel increased the secretion of IL-6 and bFGF and that both gels in general improved secretion by the cells. The point being that even though the gel with less stiffness improved both paracrine activity and retention of the EDCs, it was not sufficient to improve the treatment effect. The simple tuning of stiffness, however, was able to circumvent this, but by improving cell activity instead of retention. This underlines the necessity for increased knowledge about the mechanisms of action of the applied cell products to properly assess the impact that the hydrogel has on their abilities.

## 7. Cell Delivery Strategies

**Figure 1:**
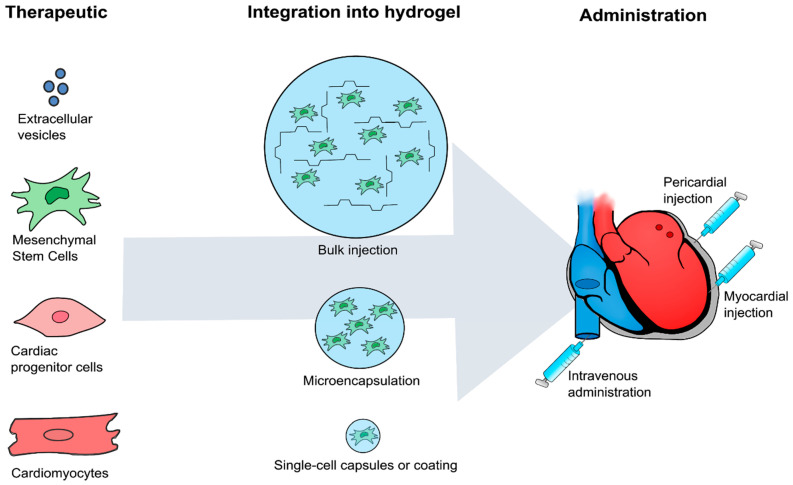
The treatment strategies reported in studies within the last four years. The most frequent therapeutic is MSCs, but EPCs, exosomes, CPCs, and differentiated cardiomyocytes have been applied likewise. The therapeutics have been integrated into hydrogels and delivered by bulk injection, microencapsulation, and single-cell capsules/coating. Combinational therapy has primarily been administered through intramyocardial injections, but intravenous and pericardial injections have also been reported.

In addition to intramyocardial delivery, which has been shown to be most effective in the clinical setting, preclinical studies have used different strategies for delivering cell-loaded hydrogels, regarding both hydrogel encapsulation and delivery route (Figure 1).

### 7.1. Bulk Delivery

The most common use of hydrogels in cell therapy has been as an injection matrix, with administrations being performed as bulk injection (Table 2). A partially cross-linked hydrogel is usually injected, and in-situ cross-linking or self-assembly solidifies the gel in the myocardium and retains the cells in this new structure [30]. The bulk delivery method can be the most straightforward approach to test since the researcher simply needs to resuspend or mix the cell suspension with the hydrogel. Issues using bulk delivery have been addressed earlier during discussion of the different clinical indications and the TESI catheter. Since even partially cross-linked hydrogels tend to be more viscous than saline, the injections themselves require more attention than using saline if applied intramyocardially. The more viscous fluid will require more time to spread throughout the myocardial fibers and can easily be ejected from the injection canal in the myocardium.

### 7.2. Microencapsulation

Another use of hydrogels for cell delivery is microencapsulation of a number of cells [12]. This method can be more demanding than bulk delivery since the cell suspension and hydrogel are often electrostatically sprayed together to form microcapsules. Another method of encapsulation is emulsion, where the cell suspension is simply mixing cell suspension with hydrogel and oil. This is an inexpensive method but with a low degree of control over capsule size, which makes it unsuitable for clinical production without extensive optimization. An increasingly popular method of overcoming the need for electrostatic spraying is to encapsulate the cells by microfluidics [67]. This provides precise control of microcapsule size and number of cells, even down to single-cell encapsulation. The use of microfluidics is more feasible to implement as part of a good manufacturing practice (GMP) production and provides a great degree of control over the final cell–hydrogel product. The specific microfluidic system depends on the hydrogel and cross-linker used and has to be optimized for the cell type of interest, since factors such as nozzle size affect cell viability and function [68]. None of the recent studies included in Table 2 used microfluidics for encapsulation. Microencapsulation has the advantage of creating physically larger cell structures, which may result in improved retention simply due to their size. Consequently, this makes the injected cells less prone to ejection once they are spread in the myocardium. In addition, the microcapsules can be injected in saline and thus do not have the same issues with viscosity as bulk delivery. Since microcapsules require hydrogels to cross-link prior to administration, there is no limit to how the cross-linking occurs, in contrast to bulk injection. Clinically, microencapsulation must be performed in a sterile environment, most likely as part of the product or immediately prior to treatment. Depending on the microencapsulation method, the prolonged time for encapsulation may be a challenge in terms of cell viability.

### 7.3. Single-Cell Coating

As an alternative to microencapsulation, single cells can also be coated with the hydrogel. This could serve to shield the cells from the environment and the immune cells but may not improve retention in terms of size or solidification. The mechanisms of retention in a single-cell coating may, therefore, not be as straightforward as with bulk delivery and microencapsulation. Gottipati et al. coated BM-MSCs with a gelatin-PEG gel and found twice the number of transplanted cells 7 days after transplantation in a mice AMI model [69]. Despite finding in vitro that the coating decreased macrophage engulfment of MSCs, there was no difference in CD68 density across treatment groups in vivo. This suggests that the improved retention was not due to improved immune evasive abilities. Clinically, the coating approach would simply require an additional step after the production of the cells, after which they could be delivered in saline using the optimized treatment workflow. For cryopreserved allogeneic treatment, this either requires the coating to be freezable, or pre-treatment mixing with the freshly thawed cell product in a sterile environment.

### 7.4. Delivery Route

Ghanti et al. delivered their cell products and cells in hydrogel microcapsules in the pericardial sac. This seems to be an attractive approach in animal studies, but this pericardial delivery method is not a part of the established clinical workflow. This diminishes the direct large-scale translatability of the study, unless such delivery methods are developed and implemented clinically. Liu et al. used hydrogel coating to initiate VEGF signaling in IV-administered MSCs, increasing their homing ability and, subsequently, their functional effect [13]. IV delivery has long been deemed inferior to both intracoronary and TESI administration. An enhanced IV approach could truly increase the clinical workflow and cost efficiency of cardiac cell therapy, and using hydrogels instead of genetic modifications, magnetic guidance, or target tissue modifications to boost the homing abilities of the cells could greatly ease the translation of this technology.

## 8. Potential Roadblocks for Clinical Translation

Despite the impressive track record of several synthetic hydrogels, they offer a number of disadvantages from a translational point of view [48,53,54,55,56,70]. These include possible complicated synthesis processes that are difficult to scale up, high cost, and toxicity inside the human body. Even with the high clinical regard of PEG and PVA, a pre-polymer such as PEG and the cross-linkers used for preparing PVA hydrogels are expensive and carrier systems based on them are, thus, not that easy to scale up. These roadblocks haunt the path of synthetic hydrogels into the clinic, and some of them gradually disappear into oblivion without ever reaching patients. For this reason, most clinically approved hydrogel carriers are based on old concepts that have been redesigned with a new furnish. To satisfy the needs of the end-users while assuring high scalability, tissue engineers improve natural and readily available biopolymers that have already been approved for clinical applications. Especially alginate and gelatin are very promising in this regard as they are easy to modify chemically and, thus, highly tunable for natural hydrogels. The natural hydrogels, however, suffer from potential batch-to-batch variation and their adaptability will always be constrained by their intrinsic properties [71,72].

In the authors’ opinion, any potential new design strategy needs to tap into these challenges one way or another to diminish the gap into the clinic. To address such challenges, next-generation carriers have already been developed, tuning natural hydrogels to generate self-healing, injectable, printable, and bioactive carrier systems. Numerous recent studies have also shown that the inclusion of multifunctional 2D nanomaterials can make them electrically active, mechanically tough, and capable of controlled drug release [73,74]. One of the reasons that the above-mentioned next-generation carrier systems have not reached the clinic yet is likely due to insufficient interactions between engineers and physicians. It is easy to design and engineer something in the laboratory, but without proper consultation with physicians and the end-user, it is highly unlikely that they ever will see the light of day in a broader and more societal context.

### 8.1. Start with the End in Mind

**Figure 2:**
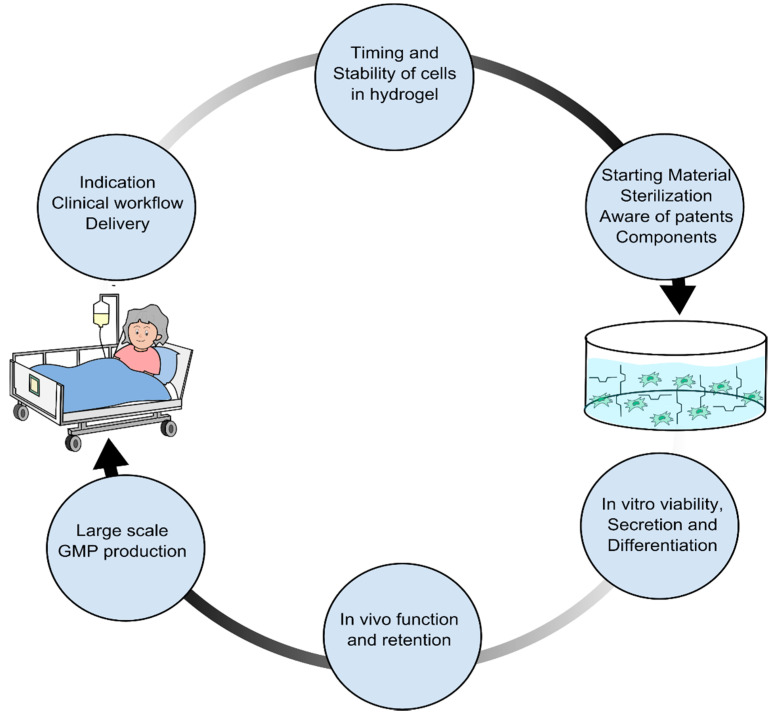
Hydrogel design process for accommodating potential roadblocks in clinical translation. Start with the clinical cell therapy workflow, indication, and workflow. Be aware of when in the cell production process or treatment workflow the cells are to be incorporated into the hydrogel. Be aware of stability after cross-linking for potential transportation between the manufacturing facility and the clinical department. Choose pure starting materials and components with proper documentation. The chosen gel type and components should be capable of being sterilized without losing their abilities and should not be covered by existing patents. When designs of hydrogel candidates are in place, the effect of the hydrogel on the specific cell product viability, secretion, and differentiation should be investigated, followed by in vivo investigation of potential functional benefits and retention.

The first thing an experienced clinician will ask when faced with new technology, such as a hydrogel, is whether it is clinically feasible to implement. It is often not. One of the fundamental issues with translational research is the lack of interaction between clinicians and basic researchers or engineers. Basic researchers and engineers aim to create the best possible hydrogel, with stiffness and motifs optimized for maximizing therapeutic effect, but they are often not aware of the clinical reality. For the hydrogel to be implemented in clinical settings, all aspects of the manufacturing for clinical use together with the logistics surrounding the treatment itself must be considered. In this case, it could be an advantage to start the design with the treatment. Since hydrogels, in our case, are for the delivery of existing cell technology, it is important to know which cell type is used, for which indication, and by which delivery system (Figure 2).

### 8.2. Treatment Scalability and Logistics

The use of freshly made hydrogel is a logistical challenge. If the hydrogel is to be produced freshly before treatment, the facility in which the treatment is being performed must have clean rooms and equipment to accommodate this. In line with this approach, a core facility could produce the hydrogel for several sites, which means that it must be stable through long-distance transportation.

Most of the studies in Table 2 applied hydrogels as bulk delivery systems for freshly cultured cells. However, a paradigm shift has occurred in the field of cell therapy, with the development from fresh autologous treatment towards cryopreserved allogeneic cell products. This affects the usability of the hydrogels. For hydrogels to be incorporated in this new clinical workflow of simply thawing the cell product prior to administration, the hydrogel must either be freezable or mixed with the cell product before administration. The last option will add extra handling of the therapeutic and requires a suited clean room and additional personnel. The issue with cryopreservation of cells in a hydrogel is that it consists of water. The crystallization during freezing will damage the cells, and incorporation of DMSO as a cryopreservative will most likely make the hydrogel fail to cross-link properly. This issue may be different for microencapsulated cells or coated cells. Here, the capsules or coated cells could be frozen in a regular cryopreservation medium and thawed when needed clinically. If hydrogels are to be used as cell delivery vehicles, these challenges must be addressed.

### 8.3. Design and Production

When deciding which type of hydrogel to develop, the hydrogel must be able to be sterilized, produced in a sterile environment, and manufactured on a large scale according to current GMP guidelines. Several hydrogels will not handle sterilization processes such as gamma irradiation, auto-cleaving, and alcohol treatment while maintaining their useful properties. This is intimately related to the fact that most polymers are highly temperature-sensitive and some of them even undergo unwanted chemical alterations after alcohol treatment or gamma irradiation. A potential method to remedy the challenges is via supercritical CO_2_ sterilization, a procedure done typically at very low temperatures to avoid possible thermal degradation issues [75]. In brief, supercritical CO_2_ sterilization is intimately linked to a critical temperature (31.1 °C) and pressure (74 atm) region that gives CO_2_ sufficient diffusivity to penetrate biomaterials completely in order to reach and inactive all possible pathogens. The fact that the procedure only relies on CO_2_ in combination with gentle temperature and pressure conditions makes it highly unlikely that biomaterials will display unwanted properties because of the sterilization process.

The polymers used for the hydrogel will need to be of utmost purity and with solid quality documentation and batch control. An example of such quality starting material is PRONOVA UP alginate, which has been used clinically as mono-treatment for AMI in the form of the IK-5001 hydrogel by Ikaria [38]. The amount of documentation and rigorous quality control is the same for all components added to the hydrogel. Over half of the hydrogels included in Table 2 have added sequences for promoting attachment, survival, or function. This makes the continuous production of these hydrogels that much more vulnerable, since the companies supplying the sequences should maintain their documentation. In addition to this, the use of some of the incorporated sequences is patented, as is the case of RGD in patent EP1894945A2, rendering the chance of successful clinical translation into practice very uncertain.

## 9. Limitations

This review has some limitations. Most of the studies investigating new therapy in cardiac indications are in AMI. The preclinical induction of AMI is not directly translatable to the clinical disease since the treatment is usually administered almost simultaneously with the onset of the AMI. Therefore, the tissue is not as soft, and intramyocardial injection is therefore feasible. This review, as all others, are potentially biased due to the general bias in the scientific literature towards positive results. Finally, for the current review, we only performed our search in PubMed, and with the following search string: ((hydrogel OR scaffold OR biomaterial) AND (“cell therapy” OR “stem cell” OR “stem cells”)) AND (cardiac OR heart). Due to the focus on stem cells in the search terms, we may not have included all relevant studies using cardiomyocytes and hydrogels.

## 10. Perspectives

We have a come a long way in recent years in terms of developing hydrogel carriers for cell therapy. Indeed, some of the carriers have already made it into clinics with promising outcomes. Most of the applied gels have shielded the cells during the injection phase and retain them inside the target site while offering them immune protection. However, in the authors’ opinion, the field has not unleashed its full potential yet. Firstly, many of the tested systems are based either on alginate, hyaluronic acid, or PEG and, therefore, are not very bioactive. Those based on bioactive collagen and gelatin tend to degrade fast from enzymatic degradation by collagenase. A way to address this in a scalable manner is, in our opinion, by incorporating gelatin into either alginate- or hyaluronic acid-based hydrogels—both of which already have been approved by the FDA—since gelatin is one of the most bioactive polymers in the field due to its naturally abundant RGD moieties. Recent in vitro studies have also shown that the inclusion of gelatin in polysaccharide-based hydrogels can increase the degradation time substantially.

If the end goal of cell and hydrogel combination therapy is complete tissue integration and continuous interaction, the formerly applied hydrogels fail by design. Conventional hydrogels are typically made from irreversible bonds which break permanently in response to cell spreading and migration, and for this reason, they are not able to withstand migration from the outside for long before breaking into pieces and dissipating inside the body. Adaptable hydrogels made from self-healable bonds could potentially yield more stable constructs in this sense, as self-healable bonds per definition are reversible and capable of reconnecting indefinitely [76]. This could be suitable for permanent cardiomyocyte integration.

Many engineers are trying to develop a cell-free cardiac hydrogel therapy. Firoozi et al. already have a strong candidate [53]. This is a next logical step in hydrogel application. However, it may be that even with a great hydrogel construct, the optimal therapy is still the combination of hydrogel and cell therapy as in most cases, there seems to be a synergistic effect [16,17,52,54,56,63].

In summary, cardiac cell therapy has great potential, and this potential can be amplified using hydrogels for delivery and shielding. Hydrogels can be tuned in many ways, but for cell therapy, the tuning must always serve to enhance the cell product. Regardless of the type and attributes of the upcoming hydrogels for cardiac cell delivery, it is our hope that the design process will begin with sparring between the engineers and the physicians.

## Figures and Tables

**Table 1:** Clinical trials using hydrogel delivery of cell therapeutics.

Study	Indication	Cell Type	Hydrogel	Application	Trial Design	ClinicalTrials.gov Identifier
**Menasché, 2018 (ESCORT)**	CABG	hESC-derived CV progenitors	Fibrin patch	Patch placed over infarct area	Open label	NCT02057900
**Chachques, 2007**	CABG	BMMNCs	Collagen scaffold	Collagen scaffold to close injection sites	Open label	N/A
**He, 2020**	CABG	UC-MSC	Collagen hydrogel	Injected cells/hydrogel co-therapy	RCT	NCT02635464

CABG: coronary artery bypass graft; ESC: embryonic stem cell; CV: cardiovascular; BMMNC: bone marrow mononuclear cells; UC-MSCs: umbilical cord-derived mesenchymal stromal cells; RCT: randomized controlled trial.

**Table 2:** Preclinical studies using hydrogel delivery of cell therapeutics since 2016.

**Study**	Indication	Cell Type	Hydrogel	Functionalization	Application	Functional Outcome Compared to Cells Only	Additional Effects
Adult Stem Cells							
**Bai, 2019**	AMI in rats	Rat BADSC	Cardiac ECM in PBS		Bulk delivery IM	Greater improvement of LVEF than BADSCs	Improved cardiac differentiation of BADSCs
**Chen, 2020**	AMI in mice	Rat AT-MSC	Transglutaminase cross-linked gelatin		Bulk delivery IM	Only group which sig. improved LVEF	Improved retention and reduced fibrosis compared to ASC in PBS, improved MTT assay, decreased ANP and TNP mRNA
**Choe, 2019**	AMI in rats	Human UC-MSC	Alginate hydrogel	Graphene oxide +/– reduction	Microencapsulation IM	No sig. improvement	Decreased fibrotic area and increased infarct thickness
**Ciuffreda, 2018**	Subacute MI in rats	Rat BM-MSC	PEG hydrogel	Heparinated	Bulk delivery IM	Only group which sig. improved LVEF	Improved retention
**Firoozi, 2020**	AMI in rats	Human BM-MSC	RADA hydrogel	SDKP peptide integration	Bulk delivery IM	Greater improvement of LVEF than BM-MSCs	Improved retention; hydrogel effect in itself better than BMMSCs; decreased fibrosis
**Follin, 2018**	AMI in immunodeficient rats	Human AT-MSC	Alginate cross-linked with calcium glucuronate		Bulk delivery IM	No difference in improvement	No difference in thickness, perfusion, or fibrosis
**Gaffey, 2018**	AMI in rats	Rat EPCs	Hyaluronate hydrogel	Adamantane and β-cyclodextrin	Bulk delivery IM	Only sig. LVEF improvement in gel + EPC group	Improved retention, myocardial velocity, and strain
**Gao, 2017**	AMI in rats	Rat BM-MSC	RADA hydrogel	SVVYGLR peptide integration	Bulk delivery IM	Greater improvement of LVEF than BM-MSCs	Decreased collagen content, infarct size, number of vessels, and decreased number of apoptotic MSCs
**Ghanta, 2020**	AMI in rats	Rat AT-MSC	TMTD alginate hydrogel		Capsules delivered epicardially	Greater improvement of LVEF than AT-MSCs	Only decreased fibrosis in the hydrogel group
**Gottipati, 2019**	AMI in mice	Mice BM-MSC	Photopolymerized gelatin methacrylamide and PEG diacrylate		Coated cells delivery IM	Improved retention by coating (double), similar macrophage density	Coating approach and no clumping tested
**Jamaiyar, 2017**	AMI in rats	Rat iVPC	PLGA microbundles		Bulk delivery IM	Only group with improved LVEF at four weeks, but no difference in improvement at eight weeks	No difference in infarct length or capillary-to-myocyte ratio
**Liu, 2017**	Acute I/R in rats	Rat BM-MSC	VEGF-gelatin-alginate-VEGF-gelatin	VEGF encapsulation	Coated cells delivered IV	Greater improvement of LVEF and perfusion than BM-MSC	Increased vascular density in peri-infarct and average area of myocardial islands in the infarct
**Qiao, 2019**	Subacute AMI in rats	Rat AT-MSCs	Porcine cardiac ECM		Bulk delivery IM	Greater improvement in LVEF compared to AT-MSCs alone	Improved hemodynamic function; increased vessel density, expression of Ang-1, and VEGF; decreased fibrosis.
**Rabbani, 2017**	AMI in rabbits	Human WJ-MSC	PEG hyaluronic acid and chitosan hydrogel		Bulk delivery IM	Greater improvement of LVEF compared to WJ-MSC	Smaller infarct area (SPECT) and increased CD31 density
**Yao, 2020**	AMI in mice	Human P-MSC	Chitosan hydrogel	IGF-1 C domain	Bulk delivery IM	Only group with sig. improved LVEF	Increased angiogenesis, reduced collagen deposition, and inhibited inflammation
**Yang, 2019**	AMI in mice	Mice BM-MSC	Chitosan thermosensitive hydrogel		Bulk delivery IM	Only group with sig. improved LVEF	Enhanced BM-MSC survival. inhibited inflammation, and alleviated pyroptosis of vascular endothelial cells
**Xu, 2017**	AMI in rats	Rat BM-MSC	Chitosan hydrogel		Bulk delivery IM	Only group with sig. improved LVEF	Improved BM-MSC retention in the myocardium
**Wang, 2020**	Chronic MI in minipigs	Human UC-MSCs	Collagen hydrogel		Bulk delivery IM	Greater improvement in cardiac output compared to UC-MSCs alone	Increased retention and myocardial tissue islands in scar tissue; decreased scar area
**Wu, 2017**	AMI in rats	Rat BM-MSC	DFEFKDFEFKYRGD small molecule hydrogel		Bulk delivery IM	Only group with sig. improved LVEF	Small-molecule hydrogel BM-MSC improved retention compared to BM-MSC alone
**Extracellular vesicles**							
**Chen, 2018**	AMI in rats	Rat EPC EVs	Hyaluronate hydrogel	Adamantane and β-cyclodextrin	Bulk delivery IM	Greater improvement in LVEF compared to EVs alone	Increased vessel density and scar thickness
**Han, 2019**	AMI in rats	Human UCMSC exosome	PA-GHRPS NapFF hydrogel	PA-GHRPS peptide and NapFF integration	Bulk delivery	Greater improvement of LVEF than exosomes	Improved retention, decreased CD68 density and TGF-b
**Lv, 2019**	AMI in rats	Rat BM-MSC derrived EV	Sodium alginate cross-linked with calcium chloride		Bulk delivery	Greater improvement of LVEF than EVs	Decreased infarct size, improved wall thickness, enhanced retention, angiogenesis, and macrophage polarization (CD206) and decreased apoptosis
**Cardiogenic cell types**							
**Bhutani, 2018**	I/R in atymic rats	Human C-kit+ CPC	PEG crosslinked with VPM	RGD or GFOGER or RDG integration	Bulk delivery	Only group with sig. improved LVEEF was non-adhesive RDG	Improved retention at day 28, decreased scar tissue
**Kanda, 2018**	Subacute MI in immunodeficient mice	Human EDC	Agarose with fibronectin and fibrinogen capsulated with PDMS		Coated cells	Greater improvement of LVEF with stiff version of gel	Improved retention. decreased scar size, and increased proliferative cardiomyocytes and non-cardiomyocytes; MMP-12, IL-6, and bFGF increased in cells in stiff gel compared to the other gel
**Tang, 2017**	AMI in mice and pigs	Human CSCs	Poly (NIPAM-AA) nanogel		Bulk delivery IM	Preserved LVEF compared to cells alone	Improved retention, prevented myocardial T cell inflammation, decreased scar size, amd increased viable myocardium and infarct thickness
**Cardiomyocytes**							
**Gerbin, 2020**	AMI in atymic rats	Human ESC-CM	Collagen gel	Notch ligand delta 1 integraion	Bulk delivery IM	Only group with sig. improved LVEF	Improved graft size
**Li, 2018**	AMI in mice	Mouse iPSC	Self-assembling folic acid modified peptide hydrogel		Bulk delivery IM	Greater improvement of LVEF than cardiac differentiated iPSC	Increased angiogenesis and decreased fibrosis

AMI: acute myocardial infarction; MI: myocardial infarction; I/R: ischemia/reperfusion; BADSC: brown adipose tissue-derived stem cells; MSC: mesenchymal stromal cell; AT: adipose tissue; BM: bone marrow; WJ: Wharton’s jelly; PL: placental; UC: umbilical cord; iVPCs: induced vascular progenitor cells; EPCs: endothelial progenitor cells; EV: extracellular vesicles; CPC: cardiac progenitor cells; EDC: Explant derived cardiac stem cells; CM: cardiomyocytes; iPSCs: induced pluripotent stem cells; ESCs: embryonic stem cells; LVEF: left ventricular ejection function; PEG: polyethylene glycol; PDMS: polydimethylsiloxane; IGF: insulin-like growth factor; IM: intramyocardial; LVEF: left ventricular ejection fraction.
